# Supplementary material for: On cross-ancestry cancer polygenic risk scores
Source: PLoS Genet. 2021 Sep 16;17(9):e1009670. doi: 10.1371/journal.pgen.1009670 (PMC8445431; doi:10.1371/journal.pgen.1009670)
Supplement: S1 Fig — (DOCX) [file pgen.1009670.s001.docx]

**S1 Fig. Observed case proportion across GWAS hits-based cancer PRS risk deciles**. Proportions of breast cancer cases (A) and prostate cancer cases (B) stratified by ancestry groups are shown. Total case counts per ancestry group are given in parentheses. Underlying sample counts and corresponding Cochran-Armitage Test for Trend P-values are reported in S3 and S4 Tables. Abbreviations: AFR: African; EAS: East Asian; EUR: European, SAS: South Asian.
