# Supplementary material for: On cross-ancestry cancer polygenic risk scores
Source: PLoS Genet. 2021 Sep 16;17(9):e1009670. doi: 10.1371/journal.pgen.1009670 (PMC8445431; doi:10.1371/journal.pgen.1009670)
Supplement: S3 Fig — (DOCX) [file pgen.1009670.s003.docx]

**S3 Fig. Prostate cancer CSPRS distributions before (A) and after (B – F) defining global risk variants.** Five sets of global variants were defined as variant whose allele frequency differences between the four ancestry groups within the 1000 Genomes Project reference were below 25% (B), 20% (C), 15% (D), 10 % (E) and 5 % (F). Red lines indicate 10% quantiles of the corresponding UKB PRS distribution in all controls. Abbreviations: AFR: African; EAS: East Asian; EUR: European, SAS: South Asian
