# Supplementary material for: On cross-ancestry cancer polygenic risk scores
Source: PLoS Genet. 2021 Sep 16;17(9):e1009670. doi: 10.1371/journal.pgen.1009670 (PMC8445431; doi:10.1371/journal.pgen.1009670)
Supplement: S5 Fig — (DOCX) [file pgen.1009670.s005.docx]

**S5 Fig. Prostate cancer CSPRS associations based on unfiltered and five global risk variant sets.** Associations are shown across the ancestry groups. Analyses were adjusted for birth year, genotyping array, and first ten principal components. Five sets of global variants were defined as variant whose allele frequency differences between the four ancestry groups within the 1000 Genomes Project reference were below 5, 10, 15, 20 and 25%. Abbreviations: AFR: African; EAS: East Asian; EUR: European, SAS: South Asian
