# Supplementary material for: On cross-ancestry cancer polygenic risk scores
Source: PLoS Genet. 2021 Sep 16;17(9):e1009670. doi: 10.1371/journal.pgen.1009670 (PMC8445431; doi:10.1371/journal.pgen.1009670)
Supplement: S7 Fig — (DOCX) [file pgen.1009670.s007.docx]

**S7 Fig.** Violin plots of the breast and prostate cancer PRS distributions. Breast cancer (left) and prostate cancer (right) C+T (CTPRS, top), Lassosum (LSPRS, middle) and LDpred (LPPRS, bottom) constructs stratified by ancestry group are shown. Black vertical lines indicate 25, 50, and 75% quantiles within the ancestry-specific case (orange) and control (green) distributions. Red lines indicate 10% quantiles of the corresponding UKB PRS distribution in all controls.
