# Supplementary material for: On cross-ancestry cancer polygenic risk scores
Source: PLoS Genet. 2021 Sep 16;17(9):e1009670. doi: 10.1371/journal.pgen.1009670 (PMC8445431; doi:10.1371/journal.pgen.1009670)
Supplement: S8 Fig — (DOCX) [file pgen.1009670.s008.docx]

**S8 Fig.** Observed case proportion across C+T-based cancer PRS (CTPRS) risk deciles
