# Supplementary material for: On cross-ancestry cancer polygenic risk scores
Source: PLoS Genet. 2021 Sep 16;17(9):e1009670. doi: 10.1371/journal.pgen.1009670 (PMC8445431; doi:10.1371/journal.pgen.1009670)
Supplement: S10 Fig — (DOCX) [file pgen.1009670.s010.docx]

**S10 Fig.** Observed case proportion across LDpred-based cancer PRS (LPPRS) risk deciles
