# Supplementary material for: On cross-ancestry cancer polygenic risk scores
Source: PLoS Genet. 2021 Sep 16;17(9):e1009670. doi: 10.1371/journal.pgen.1009670 (PMC8445431; doi:10.1371/journal.pgen.1009670)
Supplement: S11 Fig — (DOCX) [file pgen.1009670.s011.docx]

**S11 Fig**. Violin plots of the breast and prostate cancer PRS distributions in in the Michigan Genomics Initiative Study. Breast cancer (right) and prostate cancer (left) GPRS (GWAS hit-based; top) and CSPRS (PRS-CS-based PRS , bottom) stratified by ancestry group are shown. Black vertical lines indicate 25, 50, and 75% quantiles within the ancestry-specific case (orange) and control (green) distributions. Red lines indicate 10% quantiles of the corresponding MGI PRS distribution in all controls. Sample sizes for each sub-set can be found in **S7 Table**
