# Supplementary material for: On cross-ancestry cancer polygenic risk scores
Source: PLoS Genet. 2021 Sep 16;17(9):e1009670. doi: 10.1371/journal.pgen.1009670 (PMC8445431; doi:10.1371/journal.pgen.1009670)
Supplement: S12 Fig — (DOCX) [file pgen.1009670.s012.docx]

**S12 Fig**. Observed case proportion across PRS-CS-based cancer PRS risk deciles in MGI. Proportions of prostate cancer cases (A) and breast cancer cases (B) stratified by ancestry groups are shown. Total case counts per ancestry group are given in parentheses.
