# Supplementary material for: On cross-ancestry cancer polygenic risk scores
Source: PLoS Genet. 2021 Sep 16;17(9):e1009670. doi: 10.1371/journal.pgen.1009670 (PMC8445431; doi:10.1371/journal.pgen.1009670)
Supplement: S1 Table — (DOCX) [file pgen.1009670.s013.docx]

**S1 Table.** Demographics of the UK Biobank study

|  | **Ancestry Group^c^** | | | |
| --- | --- | --- | --- | --- |
|  | **EUR** | **SAS** | **AFR** | **EAS** |
| **n** | 417423 | 8103 | 6607 | 1743 |
| **Males (%)** | 189151 (45.3) | 4356 (53.8) | 2825 (42.8) | 629 (36.1) |
| **Breast Cancer (%)^a^** | 14109 (6.2) | 149 (4.0) | 116 (3.1) | 45 (4.0) |
| **Prostate Cancer (%)^b^** | 6561 (3.5) | 51 (1.2) | 144 (5.1) | 7 (1.1) |
| **Education (%)** |  |  |  |  |
| College or University degree | 134688 (32.3) | 3137 (38.9) | 2196 (33.5) | 827 (47.5) |
| A levels/AS levels or equivalent | 46984 (11.3) | 704 (8.7) | 449 (6.8) | 129 (7.4) |
| O levels/GCSEs or equivalent | 89328 (21.5) | 1291 (16.0) | 1137 (17.3) | 202 (11.6) |
| CSEs or equivalent | 22644 (5.4) | 451 (5.6) | 506 (7.7) | 44 (2.5) |
| NVQ or HND or HNC or equivalent | 27722 (6.7) | 378 (4.7) | 725 (11.1) | 78 (4.5) |
| Other professional qualifications e.g., nursing, teaching | 22345 (5.4) | 372 (4.6) | 509 (7.8) | 129 (7.4) |
| None of the above | 69081 (16.6) | 1286 (16.0) | 825 (12.6) | 233 (13.4) |
| Prefer not to answer | 3628 (0.9) | 435 (5.4) | 214 (3.3) | 98 (5.6) |
| **Income (%)** |  |  |  |  |
| Greater than 100,000 | 21016 (5.0) | 378 (4.7) | 75 (1.1) | 86 (4.9) |
| 52,000 to 100,000 | 76770 (18.4) | 1066 (13.2) | 613 (9.3) | 238 (13.7) |
| 31,000 to 51,999 | 95850 (23.0) | 1255 (15.6) | 1174 (17.9) | 343 (19.7) |
| 18,000 to 30,999 | 90801 (21.8) | 1426 (17.7) | 1501 (22.9) | 307 (17.6) |
| Less than 18,000 | 76923 (18.5) | 1915 (23.8) | 1719 (26.2) | 397 (22.8) |
| Do not know | 16180 (3.9) | 695 (8.6) | 685 (10.4) | 121 (7.0) |
| Prefer not to answer | 38880 (9.3) | 1319 (16.4) | 794 (12.1) | 248 (14.3) |

^a^ females only; ^b^ males only; ^c^ AFR: African; EAS: East Asian; EUR: European, SAS: South Asian
