# Supplementary material for: On cross-ancestry cancer polygenic risk scores
Source: PLoS Genet. 2021 Sep 16;17(9):e1009670. doi: 10.1371/journal.pgen.1009670 (PMC8445431; doi:10.1371/journal.pgen.1009670)
Supplement: S2 Table — ANOVA test was adjusted using birth year, genotyping array and first ten principal components. (DOCX) [file pgen.1009670.s014.docx]

**S2** **Table** Comparison of breast cancer and prostate cancer PRS stratified by ancestry group. ANOVA test was adjusted using birth year, genotyping array and first ten principal components.

|  | **Ancestry Group^c^** | **n** | **GPRS** | |  | **CSPRS** | |
| --- | --- | --- | --- | --- | --- | --- | --- |
|  |  |  | **Mean (s.d.)** | **ANOVA P (F-value)** |  | **Mean (s.d.)** | **ANOVA P (F-value)** |
| Breast Cancer PRS ^a^ | EUR | 228,272 | -0.0366 (0.985) | 4.6x10^-2148^ (3369.1) |  | -0.0794 (0.903) | 1.67x10^-11149^ (19114.3) |
|  | SAS | 3,747 | 0.539 (0.898) |  |  | 1.19 (0.853) |  |
|  | AFR | 3,782 | 1.37 (0.713) |  |  | 2.94 (0.74) |  |
|  | EAS | 1,114 | 1.05 (0.776) |  |  | 2.29 (0.795) |  |
| Prostate Cancer PRS ^b^ | EUR | 189,151 | -0.00601 (1.01) | 2.31x10^-141^  (218.26) |  | -0.00509 (0.99) | 3.73x10^-1229^ (1915.9) |
|  | SAS | 4,356 | -0.0145 (0.863) |  |  | -0.32 (0.923) |  |
|  | AFR | 2,825 | 0.468 (0.666) |  |  | 1.16 (0.771) |  |
|  | EAS | 629 | -0.194 (0.757) |  |  | -1.45 (0.882) |  |

Abbreviations: GWAS, genome-wide association study; PRS, polygenic risk score; PRS-CS, PRS method based on the continuous shrinkage (CS) priors; PRS, polygenic risk score; GPRS: GWAS Hits-based PRS; CSPRS: PRS-CS based PRS; s.d., standard deviation

^a^ female individuals only; ^b^ male individuals only; ^c^ AFR: African; EAS: East Asian; EUR: European, SAS: South Asian
