# Supplementary material for: On cross-ancestry cancer polygenic risk scores
Source: PLoS Genet. 2021 Sep 16;17(9):e1009670. doi: 10.1371/journal.pgen.1009670 (PMC8445431; doi:10.1371/journal.pgen.1009670)
Supplement: S3 Table — Counts by ancestry group and case-control status. (DOCX) [file pgen.1009670.s015.docx]

**S3 Table.** Breast cancer PRS risk deciles calculated within females of each ancestry group. Counts by ancestry group and case-control status.

| Ancestry Group^a^ | | Risk Decile | | | | | | | | | |  |
| --- | --- | --- | --- | --- | --- | --- | --- | --- | --- | --- | --- | --- |
|  |  | **1** | **2** | **3** | **4** | **5** | **6** | **7** | **8** | **9** | **10** | **Cochran-Armitage  Test for Trend P** |
| Breast cancer GPRS | |  |  |  |  |  |  |  |  |  |  |  |
| EUR | Cases | 588 | 800 | 903 | 1079 | 1194 | 1305 | 1460 | 1805 | 2069 | 2906 | 6.38e-551 |
|  | Controls | 21417 | 21416 | 21416 | 21416 | 21417 | 21416 | 21416 | 21416 | 21416 | 21417 |  |
| SAS | Cases | 8 | 12 | 13 | 10 | 11 | 12 | 16 | 14 | 21 | 32 | 2.45e-05 |
|  | Controls | 360 | 360 | 360 | 359 | 360 | 360 | 359 | 360 | 360 | 360 |  |
| AFR | Cases | 6 | 12 | 6 | 7 | 8 | 13 | 11 | 17 | 14 | 22 | 0.000258 |
|  | Controls | 367 | 367 | 366 | 367 | 366 | 367 | 366 | 367 | 366 | 367 |  |
| EAS | Cases | 3 | 1 | 2 | 2 | 2 | 5 | 7 | 4 | 7 | 12 | 0.000133 |
|  | Controls | 107 | 107 | 107 | 107 | 107 | 106 | 107 | 107 | 107 | 107 |  |
| Breast cancer CSPRS | |  |  |  |  |  |  |  |  |  |  |  |
| EUR | Cases | 457 | 636 | 911 | 977 | 1162 | 1310 | 1514 | 1741 | 2126 | 3275 | 6.43e-758 |
|  | Controls | 21417 | 21416 | 21416 | 21416 | 21417 | 21416 | 21416 | 21416 | 21416 | 21417 |  |
| SAS | Cases | 7 | 9 | 10 | 9 | 8 | 15 | 17 | 14 | 29 | 31 | 1.45e-08 |
|  | Controls | 360 | 360 | 360 | 359 | 360 | 360 | 359 | 360 | 360 | 360 |  |
| AFR | Cases | 9 | 5 | 7 | 4 | 5 | 10 | 13 | 18 | 17 | 28 | 5.92e-08 |
|  | Controls | 367 | 367 | 366 | 367 | 366 | 367 | 366 | 367 | 366 | 367 |  |
| EAS | Cases | 0 | 4 | 1 | 4 | 2 | 4 | 8 | 6 | 7 | 9 | 0.000342 |
|  | Controls | 107 | 107 | 107 | 107 | 107 | 106 | 107 | 107 | 107 | 107 |  |

^a^ AFR: African; EAS: East Asian; EUR: European, SAS: South Asian
