# Supplementary material for: On cross-ancestry cancer polygenic risk scores
Source: PLoS Genet. 2021 Sep 16;17(9):e1009670. doi: 10.1371/journal.pgen.1009670 (PMC8445431; doi:10.1371/journal.pgen.1009670)
Supplement: S4 Table — Counts by ancestry group and case-control status. (DOCX) [file pgen.1009670.s016.docx]

**S4 Table.** Prostate cancer PRS risk deciles calculated within males of each ancestry group. Counts by ancestry group and case-control status.

| Ancestry Group^a^ | | Risk Decile | | | | | | | | | |  |
| --- | --- | --- | --- | --- | --- | --- | --- | --- | --- | --- | --- | --- |
|  |  | **1** | **2** | **3** | **4** | **5** | **6** | **7** | **8** | **9** | **10** | **Cochran-Armitage  Test for Trend P** |
| Prostate Cancer GPRS | |  |  |  |  |  |  |  |  |  |  |  |
| EUR | Cases | 164 | 275 | 355 | 399 | 492 | 579 | 644 | 860 | 1088 | 1705 | 2.62e-507 |
|  | Controls | 18259 | 18259 | 18259 | 18259 | 18259 | 18259 | 18259 | 18259 | 18259 | 18259 |  |
| SAS | Cases | 1 | 0 | 6 | 5 | 6 | 1 | 6 | 5 | 8 | 13 | 0.000144 |
|  | Controls | 431 | 430 | 431 | 430 | 431 | 430 | 430 | 431 | 430 | 431 |  |
| AFR | Cases | 5 | 9 | 8 | 13 | 14 | 14 | 21 | 20 | 19 | 21 | 2.41e-05 |
|  | Controls | 269 | 268 | 268 | 268 | 268 | 268 | 268 | 268 | 268 | 268 |  |
| EAS | Cases | 0 | 0 | 1 | 1 | 0 | 1 | 1 | 0 | 0 | 3 | 0.134 |
|  | Controls | 63 | 62 | 62 | 62 | 62 | 62 | 62 | 62 | 62 | 63 |  |
| Prostate Cancer CSPRS | |  |  |  |  |  |  |  |  |  |  |  |
| EUR | Cases | 144 | 223 | 312 | 371 | 467 | 564 | 668 | 784 | 1092 | 1936 | 6.6e-630 |
|  | Controls | 18259 | 18259 | 18259 | 18259 | 18259 | 18259 | 18259 | 18259 | 18259 | 18259 |  |
| SAS | Cases | 1 | 1 | 0 | 1 | 3 | 4 | 8 | 4 | 13 | 16 | 2.76e-10 |
|  | Controls | 431 | 430 | 431 | 430 | 431 | 430 | 430 | 431 | 430 | 431 |  |
| AFR | Cases | 7 | 17 | 8 | 11 | 9 | 15 | 25 | 13 | 16 | 23 | 0.00297 |
|  | Controls | 269 | 268 | 268 | 268 | 268 | 268 | 268 | 268 | 268 | 268 |  |
| EAS | Cases | 0 | 0 | 1 | 1 | 0 | 1 | 0 | 0 | 1 | 3 | 0.0787 |
|  | Controls | 63 | 62 | 62 | 62 | 62 | 62 | 62 | 62 | 62 | 63 |  |

^a^ AFR: African; EAS: East Asian; EUR: European, SAS: South Asian
