# Supplementary material for: On cross-ancestry cancer polygenic risk scores
Source: PLoS Genet. 2021 Sep 16;17(9):e1009670. doi: 10.1371/journal.pgen.1009670 (PMC8445431; doi:10.1371/journal.pgen.1009670)
Supplement: S5 Table — (DOCX) [file pgen.1009670.s017.docx]

**S5 Table:** Alternative PRSs’ Performance in unrelated EUR samples of the UK Biobank Study. Methods were order by decreasing pseudo-R^2^

| **Cancer Trait: Sample Size in UKB** | **Method** | **Tuning Parameter*** | **# SNPs** | **PRS Performance** | | | **Association** | |
| --- | --- | --- | --- | --- | --- | --- | --- | --- |
|  |  |  |  | **Pseudo-R^2^** | **Brier Score** | **Adjusted AUC** | **Odds Ratio** | **P** |
| Breast Cancer:  14,109 Cases 214,164 Controls | CSPRS  (PRS-CS) | Default “auto” | 1,120,410 | 0.0460 | 0.0564 | 0.653 (0.648, 0.657) | 1.771 (1.739, 1.803) | 5.4e-857 |
|  | LSPRS  (Lassosum) | s = 0.5; l = 0.00546 | 44,576 | 0.0423 | 0.0565 | 0.645 (0.641, 0.650) | 1.710 (1.680, 1.740) | 3.5e-780 |
|  | LPPRS  (LDPred) | p(causal) = 0.03 | 6,477,992 | 0.0367 | 0.0566 | 0.637 (0.633, 0.642) | 1.670 (1.640, 1.699) | 5.4e-695 |
|  | CTPRS  (C+T) | P <= 2.50e-5 | 1,056 | 0.0357 | 0.0567 | 0.633 (0.628, 0.637) | 1.630 (1.602, 1.659) | 9.1e-661 |
|  | GPRS | P <= 5.0e-8 | 334 | 0.0329 | 0.0568 | 0.628 (0.623, 0.632) | 1.594 (1.567, 1.622) | 1.7e-613 |
| Prostate Cancer:  6,561 Cases 182,590 Controls | CSPRS  (PRS-CS) | Default “auto” | 1,120,596 | 0.0674 | 0.0316 | 0.702 (0.695, 0.708) | 2.140 (2.085, 2.197) | 4.2e-711 |
|  | LSPRS  (Lassosom) | s = 0.5; l = 0.00695 | 26,265 | 0.0652 | 0.0316 | 0.700 (0.693, 0.706) | 2.127 (2.072, 2.184) | 3.7e-692 |
|  | LDPRS  (LDPred) | p(causal) = 0.03 | 3,570,040 | 0.0583 | 0.0317 | 0.688 (0.682, 0.695) | 2.030 (1.978, 2.084) | 1.7e-618 |
|  | CTPRS  (C+T) | P <= 7.94e-5 | 1396 | 0.0570 | 0.0318 | 0.688 (0.682, 0.695) | 2.020 (1.968, 2.074) | 8.9e-610 |
|  | GPRS | P <= 5.0e-8 | 377 | 0.0521 | 0.0319 | 0.680 (0.674, 0.687) | 1.943 (1.894, 1.993) | 3.1e-566 |

* Based on evaluation in MGI cohort
