# Supplementary material for: On cross-ancestry cancer polygenic risk scores
Source: PLoS Genet. 2021 Sep 16;17(9):e1009670. doi: 10.1371/journal.pgen.1009670 (PMC8445431; doi:10.1371/journal.pgen.1009670)
Supplement: S6 Table — (DOCX) [file pgen.1009670.s018.docx]

**S6 Table.** Case enrichment in breast and prostate cancer PRS top 10% versus bottom 90% for three alternative PRS methods.

| GWAS Trait  / Outcome | Ancestry Group | CTPRS (C+T) | | LSPRS (Lassosum) | | LPPRS (LDpred) | |
| --- | --- | --- | --- | --- | --- | --- | --- |
|  |  | **OR Top 10%**  **(95% CI)** | **P** | **OR Top 10%**  **(95% CI)** | **P** | **OR Top 10%**  **(95% CI)** | **P** |
| Overall Breast Cancer | EUR | 2.45 (2.34, 2.55) | 2.81e-363 | 2.63 (2.52, 2.75) | 7.35e-437 | 2.56 (2.45, 2.67) | 2.5e-392 |
|  | SAS | 2.03 (1.32, 3.13) | 0.00137 | 2.55 (1.71, 3.80) | 4.33E-06 | 2.3 (1.52, 3.46) | 7.11E-05 |
|  | AFR | 1.98 (1.21, 3.22) | 0.00621 | 2.28 (1.44, 3.63) | 0.000482 | 2.40 (1.51, 3.82) | 0.000225 |
|  | EAS | 2.53 (1.22, 5.27) | 0.0131 | 3.17 (1.59, 6.33) | 0.00105 | 3.03 (1.52, 6.03) | 0.00165 |
| Prostate Cancer | EUR | 3.52 (3.32, 3.73) | 3.12e-388 | 3.76 (3.55, 3.98) | 2.67e-441 | 3.56 (3.36, 3.77) | 3.48e-401 |
|  | SAS | 2.77 (1.43, 5.36) | 0.00257 | 2.97 (1.58, 5.57) | 0.000711 | 5.12 (2.88, 9.10) | 2.62E-08 |
|  | AFR | 1.19 (0.68, 2.09) | 0.532 | 2.08 (1.31, 3.30) | 0.00197 | 1.69 (1.04, 2.75) | 0.0329 |
|  | EAS | 2.59 (0.49, 13.8) | 0.265 | 2.60 (0.49, 13.8) | 0.262 | 8.23 (2.00, 33.8) | 0.00348 |

Abbreviations: PRS, polygenic risk score; AFR, African; EAS, East Asian; EUR, European, SAS, South Asian
