# Supplementary material for: On cross-ancestry cancer polygenic risk scores
Source: PLoS Genet. 2021 Sep 16;17(9):e1009670. doi: 10.1371/journal.pgen.1009670 (PMC8445431; doi:10.1371/journal.pgen.1009670)
Supplement: S7 Table — (DOCX) [file pgen.1009670.s019.docx]

**S7 Table**. GPRS and CSPRS Performance in in the Michigan Genomics Initiative Study.

| **Trait** | **Method** | **Ancestry Group** | **n Cases** | **n Controls** | **PRS Association** | | **PRS Evaluation**  **AAUC**  **(95% CI)** |
| --- | --- | --- | --- | --- | --- | --- | --- |
|  |  |  |  |  | **OR (95% CI)*** | **P** |  |
| Breast Cancer | GPRS | EUR | 2993 | 16883 | 1.566 (1.503, 1.633) | 2.0E-100 | 0.621 (0.610, 0.633) |
|  |  | AFR | 157 | 1279 | 1.215 (1.019, 1.449) | 0.030 | 0.564 (0.514, 0.611) |
|  |  | EAS | 49 | 288 | 1.426 (1.039, 1.959) | 0.028 | 0.608 (0.521, 0.701) |
|  |  | SAS | 20 | 159 | 0.924 (0.587, 1.452) | 0.73 | 0.475 (0.323, 0.636) |
|  | CSPRS | EUR | 2993 | 16883 | 1.740 (1.664, 1.819) | 1.2E-132 | 0.642 (0.631, 0.654) |
|  |  | AFR | 157 | 1279 | 1.303 (1.074, 1.583) | 0.0074 | 0.557 (0.503, 0.605) |
|  |  | EAS | 49 | 288 | 1.417 (1.019, 1.972) | 0.038 | 0.604 (0.513, 0.691) |
|  |  | SAS | 20 | 159 | 1.135 (0.691, 1.863) | 0.62 | 0.533 (0.377, 0.679) |
| Prostate Cancer | GPRS | EUR | 2854 | 15070 | 1.898 (1.810, 1.991) | 1.6E-153 | 0.663 (0.651, 0.674) |
|  |  | AFR | 167 | 852 | 1.336 (1.089, 1.638) | 0.0055 | 0.587 (0.528, 0.640) |
|  |  | SAS | 12 | 190 | 1.168 (0.556, 2.454) | 0.68 | 0.536 (0.339, 0.746) |
|  |  | EAS | 7 | 280 | 1.474 (0.761, 2.854) | 0.25 | 0.721 (0.460, 0.927) |
|  | CSPRS | EUR | 2854 | 15070 | 2.004 (1.908, 2.106) | 2.5E-168 | 0.670 (0.659, 0.682) |
|  |  | AFR | 167 | 852 | 1.435 (1.161, 1.773) | 0.00082 | 0.580 (0.525, 0.634) |
|  |  | SAS | 12 | 190 | 1.357 (0.655, 2.812) | 0.41 | 0.626 (0.414, 0.822) |
|  |  | EAS | 7 | 280 | 1.453 (0.724, 2.918) | 0.29 | 0.719 (0.461, 0.913) |
