# Supplementary material for: On cross-ancestry cancer polygenic risk scores
Source: PLoS Genet. 2021 Sep 16;17(9):e1009670. doi: 10.1371/journal.pgen.1009670 (PMC8445431; doi:10.1371/journal.pgen.1009670)
Supplement: S8 Table — (DOCX) [file pgen.1009670.s020.docx]

**S8 Table**. Case enrichment in breast and prostate cancer PRS top 10% versus bottom 90% in the Michigan Genomics Initiative Study.

| **Trait** | **Ancestry Group** | **Cases** | **Controls** | **GPRS** | | **CSPRS** | |
| --- | --- | --- | --- | --- | --- | --- | --- |
|  |  |  |  | **OR (95% CI)** | **P** | **OR (95% CI)** | **P** |
| Breast Cancer | EUR | 2,993 | 16,883 | 2.21 (1.99, 2.47) | 3.8E-47 | 2.71 (2.43, 3.01) | 1.4E-73 |
|  | SAS | 20 | 159 | 0.79 (0.18, 3.38) | 0.749 | 1.10 (0.24, 5.15) | 0.902 |
|  | AFR | 157 | 1,279 | 1.42 (0.86, 2.34) | 0.173 | 1.74 (1.08, 2.81) | 0.024 |
|  | EAS | 49 | 288 | 2.12 (0.87, 5.15) | 0.0989 | 2.13 (0.83, 5.45) | 0.116 |
| Prostate Cancer | EUR | 2,854 | 15,070 | 3.15 (2.81, 3.54) | 5.3E-84 | 3.54 (3.15, 3.98) | 5.1E-101 |
|  | SAS | 12 | 190 | 3.91 (0.26, 58.3) | 0.322 | 3.05 (0.34, 27.5) | 0.32 |
|  | AFR | 167 | 852 | 1.74 (1, 3.03) | 0.0481 | 1.81 (1.02, 3.2) | 0.0425 |
|  | EAS | 7 | 280 | 2.38 (0.35, 16.1) | 0.375 | 3.64 (0.62, 21.3) | 0.152 |
