# Supplementary material for: On cross-ancestry cancer polygenic risk scores
Source: PLoS Genet. 2021 Sep 16;17(9):e1009670. doi: 10.1371/journal.pgen.1009670 (PMC8445431; doi:10.1371/journal.pgen.1009670)
Supplement: S9 Table — (DOCX) [file pgen.1009670.s021.docx]

**S9 Table.** Influence of Case-Control ratios on the association between prostate cancer and the corresponding CSPRS.

| **Ancestry Group** | **Cases** | **Controls** | **Targeted Case-Control Ratio*** | **Actual Case-Control Ratio** | **PRS Association** | |
| --- | --- | --- | --- | --- | --- | --- |
|  |  |  |  |  | **OR (95% CI)**** | **P** |
| EUR | 6,561 | 6,561 | 1:1 | 1:1 | 2.22 (2.13, 2.31) | 1.3E-332 |
|  |  | 13,122 | 1:2 | 1:2 | 2.20 (2.13, 2.28) | 7.8E-444 |
|  |  | 32,805 | 1:5 | 1:5 | 2.69 (2.10, 2.22) | 7.4E-575 |
|  |  | 65,558 | 1:10 | 1:10 | 2.16 (2.10, 2.22) | 2.2E-663 |
|  |  | 182,590 | Unmatched | 1:27.8 | 2.16 (2.10, 2.21) | 4.2E-711 |
| AFR | 144 | 144 | 1:1 | 1:1 | 1.46 (1.06, 2.01) | 0.020 |
|  |  | 288 | 1:2 | 1:2 | 1.42 (1.08, 1.86) | 0.013 |
|  |  | 627 | 1:5 | 1:4.4 | 1.39 (1.09, 1.77) | 0.0073 |
|  |  | 927 | 1:10 | 1:6.4 | 1.43 (1.13, 1.82) | 0.0027 |
|  |  | 2,681 | Unmatched | 1:18.6 | 1.44 (1.14, 1.82) | 0.0021 |
| SAS | 51 | 51 | 1:1 | 1:1 | 3.06 (1.73, 5.41) | 0.00013 |
|  |  | 102 | 1:2 | 1:2 | 4.10 (2.38, 7.05) | 3.6E-7 |
|  |  | 255 | 1:5 | 1:5 | 3.03 (2.05, 4.49) | 3.3E-8 |
|  |  | 510 | 1:10 | 1:10 | 2.56 (1.84, 3.56) | 2.2E-8 |
|  |  | 4,305 | Unmatched | 1:84.4 | 2.56 (1.92, 3.42) | 1.7E-10 |
| EAS | 7 | 7 | 1:1 | 1:1 | 0.43 (0.001, 230) | 0.79 |
|  |  | 14 | 1:2 | 1:2 | 2.44 (0.24, 25.2) | 0.45 |
|  |  | 35 | 1:5 | 1:5 | 3.06 (0.91, 10.3) | 0.07 |
|  |  | 70 | 1:10 | 1:10 | 2.10 (0.87, 5.06) | 0.097 |
|  |  | 622 | Unmatched | 1:88.9 | 2.12 (1.01, 4.48) | 0.048 |

* Maximal case control ratio when performing nearest neighbor (principal components PC1 – PC4, age at assessment) and exact matching (ancestry group, genotyping array).

** Odds ratios are given per standard deviation within ethnic group
