# Supplementary material for: On cross-ancestry cancer polygenic risk scores
Source: PLoS Genet. 2021 Sep 16;17(9):e1009670. doi: 10.1371/journal.pgen.1009670 (PMC8445431; doi:10.1371/journal.pgen.1009670)
Supplement: S10 Table — (DOCX) [file pgen.1009670.s022.docx]

**S10 Table.** Influence of Case-Control ratios on case enrichment in prostate cancer CSPRS top 10% versus bottom 90%.

| **Ancestry Group** | **Cases** | **Controls** | **Targeted Case-Control Ratio*** | **Actual Case-Control Ratio** | **Top 10% vs. Bottom 90%** | |
| --- | --- | --- | --- | --- | --- | --- |
|  |  |  |  |  | **OR (95% CI)** | **P** |
| EUR | 6,561 | 6,561 | 1:1 | 1:1 | 4.11 (3.73, 4.54) | 3.4E-172 |
|  |  | 13,122 | 1:2 | 1:2 | 4.07 (3.76, 4.40) | 7.1E-263 |
|  |  | 32,805 | 1:5 | 1:5 | 3.95 (3.70, 4.21) | 5.0E-379 |
|  |  | 65,558 | 1:10 | 1:10 | 3.98 (3.75, 4.23) | 4.1E-457 |
|  |  | 182,590 | Unmatched | 1:27.8 | 4.00 (3.78, 4.23) | 3.8E-495 |
| AFR | 144 | 144 | 1:1 | 1:1 | 1.98 (0.96, 4.08) | 0.065 |
|  |  | 288 | 1:2 | 1:2 | 2.01 (1.09, 3.69) | 0.025 |
|  |  | 627 | 1:5 | 1:4.4 | 1.71 (1.02, 2.86) | 0.043 |
|  |  | 927 | 1:10 | 1:6.4 | 1.78 (1.07, 2.94) | 0.025 |
|  |  | 2,681 | Unmatched | 1:18.6 | 1.78 (1.09, 2.92) | 0.022 |
| SAS | 51 | 51 | 1:1 | 1:1 | 4.62 (1.38, 15.5) | 0.013 |
|  |  | 102 | 1:2 | 1:2 | 7.55 (2.56, 22.3) | 2.5E-4 |
|  |  | 255 | 1:5 | 1:5 | 5.13 (2.38, 11.0) | 3.0E-5 |
|  |  | 510 | 1:10 | 1:10 | 4.39 (2.24, 8.63) | 1.7E-5 |
|  |  | 4,305 | Unmatched | 1:84.4 | 4.41 (2.43, 8.04) | 1.2E-6 |
| EAS | 7 | 7 | 1:1 | 1:1 | algorithm did not converge | |
|  |  | 14 | 1:2 | 1:2 | 370 (0.36, 386,000) | 0.095 |
|  |  | 35 | 1:5 | 1:5 | 21.0 (1.05, 419) | 0.046 |
|  |  | 70 | 1:10 | 1:10 | 6.77 (1.10, 41.6) | 0.039 |
|  |  | 622 | Unmatched | 1:88.9 | 6.53 (1.71, 25.0) | 0.0061 |

* Maximal case control ratio when performing nearest neighbor (principal components PC1 – PC4, age at assessment) and exact matching (ancestry group, genotyping array).
