# Supplementary material for: On cross-ancestry cancer polygenic risk scores
Source: PLoS Genet. 2021 Sep 16;17(9):e1009670. doi: 10.1371/journal.pgen.1009670 (PMC8445431; doi:10.1371/journal.pgen.1009670)
Supplement: S1 Text — (DOCX) [file pgen.1009670.s023.docx]

# S1 Text. Supplemental Methods

**Michigan Genomics Initiative Study**

The Michigan Genomics Initiative (MGI) Study was established as a longitudinal biorepository at the University of Michigan. Adult participants aged between 18 and 101 years at enrollment were recruited through the Michigan Medicine health system between 2012 and 2018 while awaiting diagnostic or interventional procedures either during a preoperative visit prior to the procedure or on the day of the procedure that required anesthesia. In addition to coded biosamples and secure, protected health information, participants understood that all EHR, claims, and national data sources linkable to the participant may be incorporated into the MGI databank. Each participant donated a blood sample for genetic analysis, underwent baseline vital sign testing, and completed a comprehensive history and physical assessment. The data used in this study included diagnoses coded with the Ninth and Tenth Revision of the International Statistical Classification of Diseases (ICD9 and ICD10) with clinical modifications (ICD9-CM and ICD10-CM), self-reported race and ethnicity, sex, precomputed principal components (PCs), genotyping batch, and age. Data were collected according to the Declaration of Helsinki principles [1]. MGI study participants’ consent forms and protocols were reviewed and approved by the University of Michigan Medical School Institutional Review Board (IRB ID HUM00099605 and HUM00155849). Opt-in written informed consent was obtained. Additional details about MGI can be found online (see **Web Resources**). A detailed comparison of the MGI versus UKB cohort can be found in Beesley et al, 2020 [2].

DNA from 47,364 blood samples was genotyped on customized Illumina Infinium CoreExome-24 bead arrays and subjected to various quality control filters, resulting in a set of 392,323 polymorphic variants. Principal components and ancestry were estimated by projecting all genotyped samples into the space of the principal components of the Human Genome Diversity Project reference panel using PLINK (938 individuals) [3, 4]. Pairwise kinship was assessed with the software KING [5], and the software FastIndep was used to reduce the data to a maximal subset that contained no pairs of individuals with 3rd-or closer degree relationship [6]. We removed participants without EHR data. Additional genotypes were obtained using the Haplotype Reference Consortium reference panel of the Michigan Imputation Server [7] and included over 24 million imputed variants with R^2^ ≥0.3 and minor allele frequency (MAF) ≥0.01%. Genotyping, quality control, and imputation are described in detail elsewhere [8].

Like the approach we applied to the UKB data, we used both principal component-based ancestry prediction and self-reported ethnic information to define ancestry groups. For the ancestry prediction, we applied projection into PC space of 2492 samples from the 1000 Genomes Project data as the reference and inferred the super populations membership (AFR: African, AMR: Ad Mixed American, EAS: East Asian, EUR: European, and SAS: South Asian ancestry). We combined the self-reported race and ethnicity data and the inferred super population membership to define the following four ancestry groups for downstream analyses: African (self-reported “Non-Hispanic African American” and inferred AFR), East Asian (self-reported “Non-Hispanic Asian” or East Asian and inferred EAS), European (self-reported “Non-Hispanic Caucasian” and inferred EUR), and South Asian individuals (self-reported “Non-Hispanic Asian” and inferred SAS). By doing so we excluded individuals with admixed, Hispanic, or Latin individuals and/or unknown ancestry as well as individuals where self-reported race / ethnicity did not match their inferred ancestry. For each cancer trait and each ancestry group, we extracted a maximal set of unrelated individuals (defined as kinship coefficient < 0.0884) [5] by first selecting a maximal set of unrelated cases before selecting a set of unrelated controls that was not related to any of the selected cases. [6]

We downloaded the weight files of the previously established GPRS and CSPRS constructs for breast and prostate cancer from PRSweb [9, 10] (PRSweb ID [PGS ID]: PRSWEB_PHECODE185_Pca-PRACTICAL_P_5e-08_MGI_20200608 [PGS000581], (PRSWEB_PHECODE174.1_Onco-iCOGS-Overall-BRCA_PRS-CS_MGI_20200608 [PGS000507]; and PRSWEB_PHECODE185_Pca-PRACTICAL_PRS-CS_MGI_20200608 [PGS000583]). Using the R package “Rprs” (see **Web Resources**) and the weights from the two PRS methods, the dosage-based value of each PRS was then calculated for each MGI individual. For comparability of association effect sizes corresponding to the continuous PRS across cancer traits and PRS construction methods, we centered PRS values to their mean and scaled them to have a standard deviation of 1.

**Alternative PRS methods**

We evaluated three additional PRS methods in the UKB data: LD Clumping and P-value thresholding (C + T; its PRS is denoted as CRPRS) [11], Lassosum (its PRS is denoted as LSPRS) [12], and LDpred (its PRS is denoted as LPPRS) [13]. Since each of these methods require a validation step to obtain tuning parameters, we used the data of the MGI study (see above), as a training set for the PRS construction and thus limited the discovery GWAS summary statistics to variants that overlapped with MGI and UKB (see flowchart in **S6 Fig**). Similar to the GPRS and CSPRS, we used the GWAS summary statistics made available by the “Breast Cancer Association Consortium” (BCAC) [14], and the “Prostate Cancer Association Group to Investigate Cancer Associated Alterations in the Genome” (PRACTICAL) [15] (also see **Web Resources**) both based on European ancestry samples.

For the C+T method, we performed linkage disequilibrium (LD) clumping of variants using the imputed allele dosages of 10,000 randomly selected samples and a pairwise correlation cut-off at r^2^ < 0.1 within 1Mb window. We construct many different PRS across a fine grid of p-value thresholds. The p-value threshold with the highest cross-validated pseudo-R2 in MGI was used to define the CTPRS for the UKB study. We used the software package “lassosum” [12] and 5,000 randomly selected, unrelated samples as the LD reference panel. We applied a MAF filter of 1 % and, only included autosomal variants that overlap between summary statistics, LD reference panel, and target panel. Each “lassosum” run resulted in up to 76 combinations of the elastic net tuning parameters s and λ, and consequently, in 76 SNP sets with corresponding weights used to construct 76 PRS. We then selected the PRS with the highest pseudo-R^2^ in MGI to define the LSPRS for the UKB study. In a similar manner we applied the software package “LDpred” which models various proportions of causal variants. This resulted in 13 different PRS of which we selected the PRS with the highest pseudo-R^2^ in MGI to define the LPPRS for the UKB study.

**Sensitivity analysis with limited case-control ratios in UKB**

We performed sensitivity analyses using a two-step approach: (1) determine the PRS risk deciles with the full UKB control samples and (2) limit the case control ratio to 1:1, 1:2, 1:5 and 1:10 using nearest neighbor (principal components PC1 – PC4, age at assessment) with a Mahalanobis distance with a caliper/width of 0.25 standard deviations and exact matching (ancestry group, genotyping array) implemented in the R package “MatchIt” [16]. The obtained case control studies were used to evaluate the influence of case-control ratios on PRS association and the case enrichment analysis in the tails of the PRS (see Methods).

**Supplemental References**

1. World Medical Association. World Medical Association Declaration of Helsinki: ethical principles for medical research involving human subjects. JAMA. 2013;310(20):2191-4. Epub 2013/10/22. doi: 10.1001/jama.2013.281053. PubMed PMID: 24141714.

2. Beesley LJ, Salvatore M, Fritsche LG, Pandit A, Rao A, Brummett C, et al. The emerging landscape of health research based on biobanks linked to electronic health records: Existing resources, statistical challenges, and potential opportunities. Stat Med. 2020;39(6):773-800. Epub 2019/12/21. doi: 10.1002/sim.8445. PubMed PMID: 31859414.

3. Wang C, Zhan X, Bragg-Gresham J, Kang HM, Stambolian D, Chew EY, et al. Ancestry estimation and control of population stratification for sequence-based association studies. Nat Genet. 2014;46(4):409-15. Epub 2014/03/19. doi: 10.1038/ng.2924. PubMed PMID: 24633160; PubMed Central PMCID: PMCPMC4084909.

4. Li JZ, Absher DM, Tang H, Southwick AM, Casto AM, Ramachandran S, et al. Worldwide human relationships inferred from genome-wide patterns of variation. Science. 2008;319(5866):1100-4. Epub 2008/02/23. doi: 10.1126/science.1153717. PubMed PMID: 18292342.

5. Manichaikul A, Mychaleckyj JC, Rich SS, Daly K, Sale M, Chen WM. Robust relationship inference in genome-wide association studies. Bioinformatics. 2010;26(22):2867-73. Epub 2010/10/12. doi: 10.1093/bioinformatics/btq559. PubMed PMID: 20926424; PubMed Central PMCID: PMCPMC3025716.

6. Abraham KJ, Diaz C. Identifying large sets of unrelated individuals and unrelated markers. Source Code Biol Med. 2014;9(1):6. Epub 2014/03/19. doi: 10.1186/1751-0473-9-6. PubMed PMID: 24635884; PubMed Central PMCID: PMCPMC3995366.

7. McCarthy S, Das S, Kretzschmar W, Delaneau O, Wood AR, Teumer A, et al. A reference panel of 64,976 haplotypes for genotype imputation. Nat Genet. 2016;48(10):1279-83. Epub 2016/08/23. doi: 10.1038/ng.3643. PubMed PMID: 27548312; PubMed Central PMCID: PMCPMC5388176.

8. Fritsche LG, Gruber SB, Wu Z, Schmidt EM, Zawistowski M, Moser SE, et al. Association of Polygenic Risk Scores for Multiple Cancers in a Phenome-wide Study: Results from The Michigan Genomics Initiative. Am J Hum Genet. 2018;102(6):1048-61. Epub 2018/05/22. doi: 10.1016/j.ajhg.2018.04.001. PubMed PMID: 29779563; PubMed Central PMCID: PMCPMC5992124.

9. Fritsche LG, Patil S, Beesley LJ, VandeHaar P, Salvatore M, Ma Y, et al. Cancer PRSweb: An Online Repository with Polygenic Risk Scores for Major Cancer Traits and Their Evaluation in Two Independent Biobanks. Am J Hum Genet. 2020;107(5):815-36. Epub 2020/09/30. doi: 10.1016/j.ajhg.2020.08.025. PubMed PMID: 32991828; PubMed Central PMCID: PMCPMC7675001.

10. Lambert SA, Gil L, Jupp S, Ritchie SC, Xu Y, Buniello A, et al. The Polygenic Score Catalog as an open database for reproducibility and systematic evaluation. Nat Genet. 2021;53(4):420-5. Epub 2021/03/12. doi: 10.1038/s41588-021-00783-5. PubMed PMID: 33692568.

11. International Schizophrenia Consortium, Purcell SM, Wray NR, Stone JL, Visscher PM, O'Donovan MC, et al. Common polygenic variation contributes to risk of schizophrenia and bipolar disorder. Nature. 2009;460(7256):748-52. Epub 2009/07/03. doi: 10.1038/nature08185. PubMed PMID: 19571811; PubMed Central PMCID: PMCPMC3912837.

12. Mak TSH, Porsch RM, Choi SW, Zhou X, Sham PC. Polygenic scores via penalized regression on summary statistics. Genet Epidemiol. 2017;41(6):469-80. Epub 2017/05/10. doi: 10.1002/gepi.22050. PubMed PMID: 28480976.

13. Vilhjalmsson BJ, Yang J, Finucane HK, Gusev A, Lindstrom S, Ripke S, et al. Modeling Linkage Disequilibrium Increases Accuracy of Polygenic Risk Scores. Am J Hum Genet. 2015;97(4):576-92. Epub 2015/10/03. doi: 10.1016/j.ajhg.2015.09.001. PubMed PMID: 26430803; PubMed Central PMCID: PMCPMC4596916.

14. Michailidou K, Lindstrom S, Dennis J, Beesley J, Hui S, Kar S, et al. Association analysis identifies 65 new breast cancer risk loci. Nature. 2017;551(7678):92-4. Epub 2017/10/24. doi: 10.1038/nature24284. PubMed PMID: 29059683; PubMed Central PMCID: PMCPMC5798588.

15. Schumacher FR, Al Olama AA, Berndt SI, Benlloch S, Ahmed M, Saunders EJ, et al. Association analyses of more than 140,000 men identify 63 new prostate cancer susceptibility loci. Nat Genet. 2018;50(7):928-36. Epub 2018/06/13. doi: 10.1038/s41588-018-0142-8. PubMed PMID: 29892016; PubMed Central PMCID: PMCPMC6568012.

16. Ho DE, Imai K, King G, Stuart EA. MatchIt: Nonparametric Preprocessing for Parametric Causal Inference. J Stat Softw. 2011;42(8):1-28. PubMed PMID: WOS:000292097500001.
